# Healthy lifestyle behaviors and the periodicity of mammography screening in Brazilian women

**DOI:** 10.1177/17455065211063294

**Published:** 2021-11-29

**Authors:** Ana Luísa Patrão, Maria da Conceição C. de Almeida, Sheila M Alvim Matos, Greice Menezes, Ligia Gabrielli, Emanuelle F Goes, Estela ML Aquino

**Affiliations:** 1 Center for Psychology, Faculty of Psychology and Education Science, University of Porto, Porto, Portugal; 2 Institute of Collective Health, Federal University of Bahia, Salvador, Brazil; 3 Gonçalo Moniz Institute, Oswaldo Cruz Foundation (FIOCRUZ), Salvador, Brazil; 4 Secretary of Health of the State of Bahia, Salvador, Brazil; 5 Center for Integrated Data and Information on Healthcare, Oswaldo Cruz Foundation (FIOCRUZ), Salvador, Brazil

**Keywords:** Brazilian women, Brazilian Longitudinal Study of Adult Health cohort study, health behaviors, periodicity of mammography screening

## Abstract

**Introduction:**

Certain behaviors have been associated with health promotion, including mammography screening, in women worldwide.

**Objective:**

The objective of this study was to determine whether there is an association between the periodicity of mammography screening and healthy lifestyle behaviors in Brazilian women employed at a public university in Bahia, Brazil.

**Methods:**

A total of 635 women of 50–69 years of age at the time of the interview, from the Brazilian Longitudinal Study of Adult Health cohort who were resident in Bahia, participated in the study. Data were collected using a multidimensional questionnaire that included questions on participants’ sociodemographic characteristics and health-related behaviors (smoking, alcohol consumption, leisure-time physical activity and diet) and another questionnaire that dealt with risk factors and breast cancer screening. Measures of association were calculated using simple and multivariate logistic regression.

**Results:**

The practice of physical activity, not smoking, moderate alcohol consumption and a healthy diet were the health behaviors most adopted by the women who had last had a mammogram ⩽2 years previously (which is in line with the interval recommended by the Brazilian Ministry of Health). A statistically significant association was found between a lapse of ⩾3 years since last undergoing mammography screening and excessive alcohol consumption, while a borderline association was found between the same screening interval and leisure-time physical inactivity.

**Conclusion:**

There was an association between lifestyle risk behaviors and a longer time interval between mammography screenings. The present results contribute to the debate on the use of mammography, lifestyle behaviors and health promotion among women.

## Introduction

Breast cancer is the most common form of cancer in women all over Brazil and constitutes the principal cause of death from cancer in women in the country.^1^ In the past 30 years, the number of breast cancer deaths has increased throughout Brazil.^2^ This increase is believed to be a consequence of lifestyle changes, which are compounded by the aging of the population and reflected in behaviors such as alcohol consumption and physical inactivity as well as postmenopausal obesity, older age at first pregnancy, low parity and breastfeeding for shorter periods of time.^3–5^ Early detection and treatment of breast cancer are considered the principal means of reducing the risk of death from this condition.^6,7^

Mammography is the procedure of choice for breast cancer screening, although the effectiveness of the method is relatively low, accounting for no more than a 20%–30% reduction in deaths from breast cancer in settings in which screening, diagnosis and treatment are implemented in an organized fashion.^6,8^ Nevertheless, there is currently an ongoing, highly pertinent debate on the adverse effects of mammography screening, namely, with respect to false-positives, over-diagnosis and over-treatment, as well as the cancers caused by ionizing radiation.^6–10^ Therefore, it is important to increase understanding on the factors associated with regular mammography screening, particularly those related to lifestyle and health promotion.

Given their role in the onset and progression of current chronic diseases, the practice of physical activity, smoking, alcohol consumption and diet are the health behaviors most associated with a healthy lifestyle.^11–13^ Furthermore, they are beginning to be reported as indicators of other behaviors linked to lifestyle^14,15^ and have also been thoroughly investigated in the field of breast cancer prevention.^16,17^

More recently, these behaviors have been associated with the periodicity of breast cancer screening in women worldwide.^18–30^ In general, those studies have pointed to a trend toward health promotion and a greater commitment to healthy behaviors in women who undergo mammography screening at more regular intervals, while a greater association has been found between risk behaviors and a longer time interval between mammography screenings. Since this is a pertinent topic and one that has been the subject of little research in Brazil, the objective of the present study was to determine whether there is an association between the periodicity of mammography screening and healthy lifestyle behaviors in women participating in the Brazilian Longitudinal Study of Adult Health (ELSA-Brasil) cohort study.

## Methods and materials

### Study design and population

The present cross-sectional study reports on the results of a supplementary study nested within the ELSA-Brasil^31^ titled “Healthcare, Classic Risk Factors and Early Breast Cancer Detection Among Workers at a Public University in Bahia.” The ELSA-Brasil sample size estimate was based on the main study outcomes: myocardial infarction and type 2 diabetes. Since the incidence of diabetes in the Brazilian population is unknown, a conservative 3-year cumulative incidence of 1.4% has been estimated. Considering an alpha value of 5%, statistical power of 80%, prevalence of exposure of 20% and relative risk of 2.0, the necessary sample size was estimated at approximately 6400 individuals. This sample size would also allow for an adequate number of incident myocardial infarctions, as the estimated incidence of myocardial infarction, based on mortality data, is expected to be slightly higher than that of diabetes. To present gender-specific analyses and allow for possible loss to follow-up, the sample size was defined at approximately 15,000 people. Women aged between 50 and 69 years, ELSA-Brasil participants and residents of the State of Bahia, who agreed to participate in the research, were eligible for the supplementary study.^31,32^ One of the objectives of that supplementary study was to investigate the pattern of use of public and private health care services with respect to gynecological care, clinical examinations and imaging tests (mammography and ultrasonography).

With the participation of 15,105 active or retired civil servants at baseline and involving six public institutes of higher learning and research in the northeast, south and southeast of Brazil, the ELSA-Brasil is the largest cohort study to be conducted in Latin America. A total of 8218 of these participants were women. Active and retired women living in Bahia who were part of the original cohort and agreed to participate in the study were included in the supplementary study on breast cancer. Women of 50–69 years of age at the time of the interview who had no cognitive and/or physical deficiencies that would prevent them from providing the information of interest were eligible for inclusion. Participants of the ELSA-Brasil cohort who were very ill or who had moved away from Salvador were excluded from the study, leaving 771 eligible women. The present analysis further excluded 102 women who were unable to provide information on the date of their last mammogram; 19 women who self-reported their ethnicity as yellow or Indigenous—in view of their small number and the impossibility of grouping them together in another category due to the specificities involved; and 15 women who failed to answer the question on ethnicity. Consequently, the final analysis included a total of 635 women.

### Measures

The data from the supplementary study were linked to the data produced at baseline of the main ELSA-Brasil study (2008–2010) and the data produced 4 years later (wave 2: 2012–2014). Sociodemographic (wave 1) and lifestyle-related variables (wave 2) come from the ELSA-Brasil questionnaires. The ELSA-Brasil instruments were duly adapted and previously published.^33^ Only “periodicity of mammography screening” comes from the supplementary study, developed between October 2014 and April 2015. These data were obtained at interviews using a questionnaire adapted from the standardized instrument developed as part of the International Pooling Project of Mammographic Density^34^ (http://mdpool.iarc.fr/). The items from this instrument were translated and adapted into Portuguese. Questions related to the prior use of health services for early detection and treatment of breast cancer were raised from the National Health Survey.^35^ The variables under investigation were the following:

*Age:* Calculated from the participant’s date of birth.
*Ethnicity/skin color:* Self-reported according to the categories used in the Brazilian census: black, brown or white.
*Education:* Data coded in accordance with the participant’s answer to the question “Up to what school level did you study?”
*Social class:* Evaluated as low, middle or high. This is a compound indicator based on the type of work the individual performs/performed (classified according to the Brazilian Classification of Occupations—CBO), whether the individual worked in a supervisory and/or management capacity, and education level.
*Marital status:* Based on answers to the question “What is your current marital status?”
*Current smoking:* Based on answers to the question “Do you currently smoke cigarettes?” (yes/no).
*Alcohol consumption:* Excessive alcohol consumption was defined based on answers to detailed questions on weekly consumption of alcoholic beverages (red and white wine, draught and bottled beer, and distilled spirits) and classified as excessive or not (<140 grams/week = not excessive; ⩾140 grams/week = excessive) in accordance with the recommendations of Duncan et al.^36^ An example of one of the questions is “How many glasses of red wine do you drink per week?”
*Leisure-time physical activity:* Measured according to the International Physical Activity Questionnaire (IPAQ).^37^ Participants reporting ⩾150 min of walking or moderate physical activity per week or ⩾60 min of strenuous physical activity per week were classified as physically active, while those reporting <150 min of walking or moderate physical activity per week or <60 min of strenuous physical activity per week were considered physically inactive. Moderate physical activity includes swimming or pedaling at a moderate pace, taking part in sports for pleasure and so on, while strenuous activity includes running, working out in a gym, pedaling fast, taking part in competitive sports and so on. An example of one of the questions is, “On how many days of the week do you walk in your leisure time?”
*Diet (consumption of fruit, vegetables and legumes):* Evaluated based on self-reports from the participants at baseline regarding their daily consumption of fruit, vegetables and legumes. Participants were asked, “How often do you usually eat raw or cooked vegetables and legumes, not including potatoes, cassava or yam?” and “How often do you usually eat fruit, not including fruit juices?” In addition, changes in diet between the baseline evaluation (wave 1) and that conducted 4 years later (wave 2) were evaluated with respect to the consumption of these dietary items based on the question “In the past 6 months have you changed your dietary habits or are you on a diet for any reason?” The reasons given include measures toward a healthier diet such as reducing cholesterol or salt intake, measures to control diabetes, lose weight and so on.
*Periodicity of mammography screening:* Based on the question “When was the last time you had a mammogram?” The answers were dichotomized as ⩽2 years or ⩾3 years.

### Ethical considerations

All the participants of this study signed an informed consent form. The ELSA-Brasil protocol was submitted to and approved by the internal review boards of the participating institutes and by the Ministry of Health’s National Commission for Ethics in Research (CONEP) (Reference—CAAE: 30614714.8.1001.5030).

### Data analysis

The frequency distribution of the independent variables (sociodemographic variables and those related to health and lifestyle) was calculated according to the time elapsed since the last mammogram. Having undergone mammography screening ⩾3 years before was considered the endpoint, and unhealthy behaviors, that is being physically inactive, excessive alcohol consumption, an unhealthy diet and being a current smoker, constituted the exposure factors. The association between the endpoint and each exposure factor examined was analyzed using unconditional logistic regression. The co-variables with p-values < 0.20 in the stratified analysis were selected for inclusion in the models.^38^ The odds ratios (ORs) and their respective 95% confidence intervals (95% CIs) were calculated as measures of association. Interactions were evaluated for the two models by introducing product terms. Next, the co-variables selected as possible confounding factors were tested, comparing the differences between the measures of association of the complete model and those adjusted for each co-variable. The STATA statistical software program, version 13, was used throughout the statistical analysis.

## Results

The only sociodemographic factors found to be associated with having undergone mammography screening ⩾3 years previously, that is, with an interval greater than that recommended by the Ministry of Health, were marital status and having private health care insurance. A greater percentage of the women who had undergone mammography screening ⩾3 years previously had no stable partner (10.6% vs 4.1%) and no private health insurance (24.7% vs 5.1%) compared to those who had undergone screening within the recommended interval. Furthermore, inadequate mammography screening was found to be associated with two characteristics related to lifestyle: being physically inactive (4.6% vs 9.0%) and excessive alcohol consumption (7.1% vs 17.5%) (Table 1).

**Table 1.** Sociodemographic, health-related and lifestyle-related characteristics of the participants according to the time elapsed since their last mammogram. ELSA-Brasil 2012–2014.

Characteristics	Time since last mammogram				p value
	⩽2 years		⩾3 years		
	n = 585	%	n = 50	%	
Sociodemographic data					
Age group					0.145
50–59 years	320	93.6	22	6.4	
⩾60 years	265	90.4	28	9.6	
Ethnicity/skin color					0.842
Black	225	92.6	18	7.4	
Brown	256	91.4	24	8.6	
White	104	92.9	8	7.1	
Education level					0.411
University	305	93.0	23	7.0	
High school	270	91.2	26	8.8	
Social class					0.132
Upper	149	94.9	8	5.1	
Lower/middle	433	91.2	42	8.8	
Marital status					0.003
Married/stable partner	258	95.9	11	4.1	
Unmarried/no stable partner	312	89.4	37	10.6	
Health-related factors					
Private health care insurance					0.000
Yes	508	94.9	27	5.1	
No	67	75.3	22	24.7	
Lifestyle-related factors					
Leisure-time physical activity					0.058
Yes	185	95.4	9	4.6	
No	384	91.0	38	9.0	
Type of diet					0.998
Healthier	433	92.1	37	7.9	
Less healthy	152	92.1	13	7.9	
Smoker					0.316
Yes	28	87.5	4	12.5	
No	547	92.4	45	7.6	
Excessive alcohol consumption					0.017
Yes	33	82.5	7	17.5	
No	537	92.9	41	7.1	

Having last undergone mammography screening ⩾3 years previously was positively associated with excessive alcohol consumption (OR = 2.77; 95% CI: 1.16–6.66). This association persisted, even after independent adjustment for each one of the co-variables selected. Conversely, the statistical significance of the association between the time elapsed since the last mammography screening and physical inactivity was only borderline (OR = 2.03; 95% CI: 0.96–4.29) (Table 2).

**Table 2.** Association between having had last mammogram ⩾3 years previously and leisure-time physical inactivity and excessive alcohol consumption—unadjusted and following adjustment for selected co-variables.

Co-variables	Last mammogram ⩾3 years previously and leisure-time physical inactivity		Last mammogram ⩾3 years previously and excessive alcohol consumption	
	OR	95% CI	OR	95% CI
Unadjusted OR	2.03	0.96–4.29	2.77	1.16–6.66*
Adjusted OR				
Age group^a^				
50–59 years	1.00	–	1.00	–
⩾60 years	2.08	0.98–4.40	2.97	1.22–7.19
Social class^a^				
Upper	1.00	–	1.00	–
Lower/middle	1.92	0.90–4.08	2.61	1.08–6.31
Marital status^a^				
Married/stable partner	1.00	–	1.00	–
Unmarried/no stable partner	2.02	0.95–4.28	2.81	1.15–6.83
Private health care insurance^a^				
Yes	1.00	–	1.00	–
No	1.68	0.78–3.63	2.79	1.10–7.07

OR: odds ratio; CI: confidence interval.

a Adjusted for each one of the co-variables independently.

* p<0.05.

For theoretical reasons, it was decided to maintain the practice of leisure-time physical activity as an exposure factor in the model. In the evaluation of the interaction and confounding variables, neither effect modifiers nor confounders were confirmed in this model (Table 3).

**Table 3.** Final models of the association between having had last mammogram ⩾3 years previously and leisure-time physical inactivity and excessive alcohol consumption.

	OR	95% CI
Mammography ⩾3 years previously and leisure-time physical inactivity^a^	2.03	0.96–4.29
Mammography ⩾3 years previously and excessive alcohol consumption^a^	2.77	1.16–6.66*

OR: odds ratio; CI: confidence interval.

a No effect modifiers or confounders were confirmed in this model.

* p<0.05.

## Discussion

The women in the present study whose last mammogram was performed ⩾3 years previously were more likely to be unmarried/have no stable partner and to have no private health insurance. These findings are to be expected in Brazil, a setting where the public health care system (SUS) has a relatively recent history of breast cancer screening compared to high-income countries and is still unable to provide this service with the desired quality to all women.^2,6^ Furthermore, the private health care system has a very strong presence despite only being within the reach of women who are better off socioeconomically.^39^

The women who had not been screened within the timespan recommended by the Ministry of Health are those with the least healthy lifestyle, that is, they are less physically active and more likely to drink excessively, although there was no difference with respect to diet or smoking. These results are in agreement with data from other studies showing that women with a healthier lifestyle in general are more likely to undergo regular mammography screening and to comply with a scheduled mammogram appointment.^18,23,25,27,30^ Likewise, not attending appointments or not undergoing breast cancer screening are behaviors associated with risky health-related behaviors.^24^ Oran et al.^30^ studied Turkish women from academic institutes and found that breast and cervical cancer screening was significantly associated with a healthier lifestyle, with results highlighting a subgroup of women more committed to health-promoting behaviors. Kim et al.^40^ also found that perceiving benefits in committing to health-promoting behaviors was a predictor of compliance with mammography screening in Chinese women.

Unlike the other lifestyle-related behaviors, most studies on alcohol consumption are not in agreement with the present study, with previous findings showing that the women who consume more alcohol are those who are most committed to attending mammography screening at regular intervals.^25,41,42^ This could be because alcohol is a known risk factor for breast cancer,^43,44^ meaning that women who drink excessively realize that they are at a greater risk of the disease and are therefore more likely to seek diagnostic centers. Nevertheless, some other studies^20^ reached conclusions that were similar to those of the present study: a longer time since mammography screening was associated with high-risk alcohol consumption. Given the controversy with respect to this particular behavior, it is considered of the utmost importance to evaluate this question at greater depth in the next waves of the ELSA-Brasil to determine whether excessive alcohol consumption remains associated with a longer mammography screening interval in the present sample.

Regarding physical activity, the present results are in agreement with the findings of various other studies. A nationwide study found that Brazilian women who had not undergone mammography screening were more likely to self-evaluate their state of health as negative (poor or very poor) and were less likely to regularly practice physical activity.^19^ The study conducted by Lagerlund et al.^25^ with a cohort of Swedish women concluded that, in general, those less committed to mammography screening were, among other aspects, less physically active outside the work environment. Furthermore, Maxwell et al.^29^ reported that, in Canadian women, one of the predictors of never having undergone mammography screening was being physically inactive or performing little physical activity. Conversely, in Taiwan, Lin^27^ found that commitment to physical activity had a positive effect on compliance with mammography screening.

Although in the present study no association was found between smoking and the periodicity of mammography screening, several studies have reported smoking as being one of the factors most commonly present in the association between lifestyle and attending mammography screening,^18,23,25,27,29^ including in Brazil,^21^ suggesting that this factor needs to be investigated in greater depth in future studies to be conducted with the present population. It should be emphasized that few of the participants in the ELSA-Brasil smoked, with smoking being particularly uncommon among the participants from Salvador who comprise the present sample. Regarding diet, few studies have been conducted on this subject and no association has been found between this behavior and the periodicity of mammography screening, although some studies have reported an association between less frequent mammography screening and obesity.^28^

In summary, the present results support the hypothesis that, in general, women with less healthy lifestyles insofar as alcohol consumption and physical inactivity are concerned are less committed to attending regular mammography screening. On the contrary, it is reasonable to assume that attending mammography screening regularly is part of a general health promotion plan in women more committed to a healthy lifestyle. Therefore, it is important to clarify and call particular attention to the fact that if, on one hand, mammography screening is associated with the greater survival of women worldwide, on the other hand, there are also negative effects (false-positives, over-diagnosis and over-treatment, as well as cancers induced by ionizing radiation).^6,10^ Furthermore, mammography screening does not prevent breast cancer, as is often conveyed in breast cancer prevention and awareness campaigns. In other words, it is important that these women who are more committed to a healthy lifestyle are duly informed with respect to the negative aspects associated with mammography screening and not only the positive effects, as more generally communicated by health care professionals.

The women who participated in this study are civil servants who volunteered to take part; therefore, any generalization of these results should be made with caution. In addition, the factors evaluated here were self-reported by the participants in all cases and are therefore subject to memory and social-desirability biases. Therefore, future studies could benefit from the use of more objective markers such as the inclusion of the mammography report or a written log of the number of alcoholic beverages ingested per week/month. Another aspect is that we cannot be sure that the women who had a mammogram a shorter time ago (⩽2 years) actually attend this type of screening more regularly, since the data obtained are restricted to the last mammogram. Nevertheless, those whose last mammogram was ⩾3 years previously would appear to undergo screening less frequently and not within the recommended interval. It is important to note that the operational definitions of healthy and risky lifestyle behaviors differ across studies so direct comparisons with previous studies can be tricky. However, despite these possible differences, the trend toward health or risk of these behaviors is clear. On the contrary, the strongpoints of the present study include the fact that it is innovative in Brazil, a middle-income country where factors involved in breast cancer screening remain relatively unknown. Since this is a working cohort, several of the aspects dealt with here could be investigated in greater depth in a longitudinal data analysis and with the incorporation of new variables that would add to the complexity of the explanation on the health behaviors presently studied.

## Conclusion

The women who were less committed to attending mammography screening were also those more likely to consume alcohol excessively and to be less physically active, suggesting that attending mammography screening and healthy lifestyle behaviors are interlinked, in such a way that there is an association between risk behaviors and a longer time interval between mammography screenings. Specifically, these results allow for a better understanding of the profile of women in terms of health, which may better guide the recommendations given by health promotion professionals and also by mammographic screening services.

Although the benefits of mammography screening in terms of the early diagnosis and treatment of breast cancer have to be taken into account, the fact is that screening does not prevent breast cancer. Although essential for early diagnosis and treatment, mammography screening is not a preventive health behavior (unlike other behaviors associated with a healthy lifestyle) but, rather, a diagnostic tool. The present study may contribute toward improving knowledge on the level of use of mammography screening and lifestyle health behaviors among Brazilian Women.
